# Utility of the Measurement of Carboxyhemoglobin Level at the Site of Acute Carbon Monoxide Poisoning in Rural Areas

**DOI:** 10.1155/2016/6192369

**Published:** 2016-04-30

**Authors:** Makoto Onodera, Yasuhisa Fujino, Satoshi Kikuchi, Masayuki Sato, Kiyofumi Mori, Takaaki Beppu, Yoshihiro Inoue

**Affiliations:** ^1^Department of Critical Care Medicine, Iwate Medical University, Morioka 020-8505, Japan; ^2^Department of Neurosurgery, Iwate Medical University, Morioka 020-8505, Japan

## Abstract

*Objective*. This study examined the hypothesis that correlations exist between the carbon monoxide exposure time and the carboxyhemoglobin concentration at the site of carbon monoxide poisoning, using a pulse carbon monoxide oximeter in rural areas or the carboxyhemoglobin concentration measured at a given medical institution.* Background*. In previous studies, no definitive relationships between the arterial blood carboxyhemoglobin level and the severity of carbon monoxide poisoning have been observed.* Method*. The subjects included patients treated for acute carbon monoxide poisoning in whom a medical emergency team was able to measure the carboxyhemoglobin level at the site of poisoning. We examined the relationship between the carboxyhemoglobin level at the site of poisoning and carbon monoxide exposure time and the relationships between the arterial blood carboxyhemoglobin level and carbon monoxide exposure time.* Results*. A total of 10 patients met the above criteria. The carboxyhemoglobin levels at the site of poisoning were significantly and positively correlated with the exposure time (rs = 0.710, *p* = 0.021), but the arterial blood carboxyhemoglobin levels were not correlated with the exposure time.* Conclusion*. In rural areas, the carboxyhemoglobin level measured at the site of carbon monoxide poisoning correlated with the exposure time.

## 1. Introduction

The diagnosis of carbon monoxide (CO) poisoning is determined based on the patient's history of CO exposure, symptoms, and arterial blood carboxyhemoglobin (CO-Hb) concentration. Although previous studies have assessed the relationship between the CO-Hb level and the severity of CO poisoning, no definitive relationships have been observed [1–4]. The CO-Hb level decreased over time as the patient is moved away from the site of poisoning and receives oxygen from the emergency medical team [4]. Therefore, especially in rural areas, the CO-Hb level cannot be said to accurately reflect the prognosis or severity of CO poisoning. In recent years, a pulse carbon monoxide oximeter (Rad-57, Masimo, Inc., Irvine, California, USA) was developed to noninvasively measure the blood CO concentration and has been shown to be useful in prehospital and emergency department (ED) screening for CO poisoning [5, 6]. However, there have been no previous reports regarding the relationship between the CO-Hb concentration (SpCO) at the site of CO poisoning and the CO-Hb concentration upon arrival to the medical institution. This study examined the hypothesis that correlations exist between the CO exposure time and either the SpCO level or the CO-Hb concentration measured at a given medical institution.

## 2. Methods

This retrospective cohort study was conducted in acute CO poisoning patients that had been urgently transported to our advanced critical care and emergency center between January 2012 and March 2014. We examined the patients in whom a medical emergency team was able to measure the SpCO level at the site of poisoning. We measured the SpCO level, CO exposure time, transport time from the site of poisoning to the medical institution, and CO-Hb level measured at the medical institution. An assessment was made of the relationship between the SpCO level and exposure time and the relationships between the CO-Hb and exposure time.

In five areas of Iwate prefecture, ambulances belonging to fire department headquarters were equipped with pulse CO-oximeters (Rad-57, Masimo Japan, Inc., Tokyo, Japan). The five areas included northern Morioka City, Hachimantai City, Iwate Town, Kuzumaki Town, and Shizukuishi Town, all of which are located over 25 km away from our institution. The SpCO levels were obtained by evaluating the index finger after it had been disinfected. All patients underwent normobaric oxygen therapy during transportation from the site of CO poisoning to our institution. The CO-Hb measurements were obtained using a blood gas analyzer (RAPIDLab 1265 Systems, Siemens Japan, Co., Ltd., Tokyo, Japan), and hyperbaric oxygen therapy (HBOT) was performed in cases where the CO-Hb level was 25% or greater (a Colignon and Lamy criterion [7]), as well as in cases where syncope was observed in the field despite a CO-Hb level of less than 25%. It was also performed in cases where the brain CT/MRI revealed abnormal findings in globus pallidus and cerebral white matter (e.g., the brain CT revealed low density area or the brain MRI revealed a high signal in T1-weighted imaging and a low signal in T2-weighted imaging).

### 2.1. Statistical Analysis

The values are expressed as the mean ± standard deviation. A correlation analysis was performed using Spearman's rank correlation. The significance level (*p* < 0.05) was set. In addition, a single regression analysis was carried out according to the least squares method. All statistical analyses were performed using the SISS software program (ver. 2012 SISS for Windows, Tokyo).

## 3. Results

### 3.1. Patients

Among the acute CO poisoning cases that occurred within the aforementioned period, the emergency services sent their pulse CO-oximeters to our hospital in 10 cases (20%). The other 80% of cases occurred in areas where pulse CO-oximeters were not used. The patients consisted of six males and four females, with a mean age of 49.4 ± 21.4 years. CO poisoning involved accidents related to the use of white gas or briquette coal in seven patients, inhalation burns due to fire in two patients, and severe burns due to self-immolation in one patient. The outcome was survival in nine patients and death in one patient with severe burns. Loss of consciousness at the site of CO poisoning occurred in two patients. The level of consciousness at the time of patient contact was 15 points on the Glasgow Coma Scale in nine patients and 14 points in one patient. HBOT was performed in four patients.

### 3.2. SpCO

**Figure 1:**
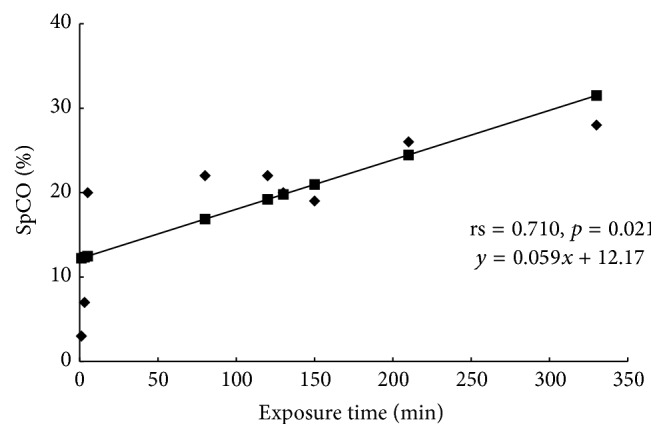
CO-Hb levels (SpCO) measured at the site of CO poisoning using a pulse CO-oximeter versus the CO exposure time.

The SpCO level was 18.9 ± 7.9%, and the exposure time was 115.1 ± 102.8 minutes. There was a significant and positive correlation between the SpCO level and the exposure time (rs = 0.710, *p* = 0.021), and the regression line was as follows: *y* = 0.059*x* + 12.17 (Figure 1).

### 3.3. Blood CO-Hb

**Figure 2:**
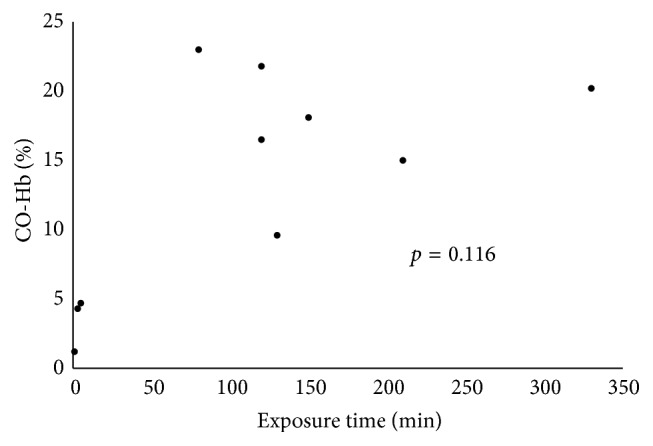
Laboratory blood gas analysis data (CO-Hb) versus the exposure time.

The transport time was 76.2 ± 30.5 minutes and the blood CO-Hb level was 13.4 ± 7.9%. There were no correlations between the blood CO-Hb level and the exposure time (*p* = 0.116) (Figure 2).

## 4. Discussion

In this study, the SpCO level was found to correlate with the exposure time at the site of CO poisoning in rural areas. Our findings appear in this respect to be consistent with previous findings. Lam et al. [8] examined 78 subjects living in Guatemalan Highlands communities who used wood-fired* temazcal* (sauna bath) and measured the SpCO levels before and after the* temazcal* use. The CO-Hb level was calculated based on ppm. The authors subsequently reported a positive correlation between the CO-Hb level and the length of time of* temazcal* use. Additionally, Topacoglu et al. [9] examined 20 male volunteers who worked at parking lot and carwash facilities and measured the SpCO levels before and after work and concluded that the mean CO-Hb level significantly increased due to the exhaust fumes. Another study measured the SpCO levels in subjects in a bingo hall in which smoking was permitted [10], and other studies have measured the SpCO levels in children exposed to parental smoking and those without such exposure [11, 12]. No statistically significant differences in the SpCO measurements were found in these studies, although the aforementioned studies showed a relationship between the SpCO level and exposure time when there was a high CO concentration in the environment. Therefore, we posit that SpCO level is useful in assessing the severity of acute CO exposure.

Studies conducted to date have examined the relationship between the SpCO and CO-Hb levels [13–15]. One such study was that of Piatkowski et al. [15], who examined 20 patients with CO poisoning. Their findings showed the usefulness of pulse CO-oximeters for obtaining an early diagnosis of CO poisoning. Furthermore, Kot et al. [13] examined 49 cases of CO poisoning and found a strong positive correlation between the CO-Hb and SpCO levels. However, these two studies involved simultaneous measurements of the CO-Hb and SpCO level obtained at medical institutions. Hence, the relationship between the SpCO level measured at the site of CO poisoning and the CO-Hb level measured at a medical institution is unclear. The present study examined the relationship between these two parameters and the exposure time at the site of CO poisoning and found a significant and positive correlation between the SpCO level and the exposure time.

We predicted that the CO-Hb levels would be higher in the patients with a long exposure time. However, the present findings showed that the CO-Hb level was not correlated with the exposure time. Previous studies have reported that the CO-Hb level does not correspond with the severity of CO poisoning [4, 14, 16]. The most likely reason for this finding is that the CO-Hb level decreases as the patient is moved away from the site of CO poisoning and receives oxygen from the emergency medical team [4]. In the current study, a mean of transport time was 76.2 minutes. Hence, this result is thought to be due to the change in the CO-Hb level as a result of the administration of high-flow oxygen by the emergency medical team.

Some limitations are associated with the present study. Firstly, only one institution was involved, and the number of cases was small. Secondly, the subjects included patients with inhalation burns due to fire and severe burns due to self-immolation. In addition, there are various opinions regarding the precision of the CO-oximeter [17–19], and no consensus has been obtained. Going forward, a comparison will probably be necessary between the blood CO-Hb and SpCO values that arise in the field.

## 5. Conclusions

The SpCO level was found to correlate with the exposure time at the site of CO poisoning in rural areas. In-field SpCO measurement is rapid and repeatable and may be useful in gaining an understanding of the extent of exposure in the critical early moments of medical response. However, at this time further direct correlation with CO-Hb levels as measured in the hospital is limited, and further study is warranted.
